# Mechanistic analysis of Tanshinone IIA’s regulation of the ATM/GADD45/ORC signaling pathway to reduce myocardial ischemia-reperfusion injury

**DOI:** 10.3389/fphar.2024.1510380

**Published:** 2024-12-24

**Authors:** Yiwei Sang, Jiangnan Du, Dilimulati Zulikala, Zhongqiang Sang

**Affiliations:** ^1^ Nature Drug Discovery Group, School of Pharmacy, Queen’s University Belfast, Belfast, United Kingdom; ^2^ Dermatology Department, Shanghai Zhongye Hospital, Shanghai, China

**Keywords:** myocardial ischemia-reperfusion injury, H9C2, ATM, GADD45, ORC

## Abstract

**Background:**

By far, one of the best treatments for myocardial ischemia is reperfusion therapy. The primary liposoluble component of Danshen, a traditional Chinese herbal remedy, Tanshinone ⅡA, has been shown to have cardiac healing properties. The purpose of this work is to investigate the processes by which Tanshinone ⅡA influences myocardial ischemia-reperfusion injury (MIRI) in the H9C2 cardiac myoblast cell line, as well as the association between Tanshinone ⅡA and MIRI.

**Methods and results:**

The cardiac cells were divided into a normal group, a model group and Tanshinone ⅡA treatment groups. After 4 h of culture with the deprivation of oxygen and glucose, the cells were incubated normally for 2 h. The success of the model and the capacity of Tanshinone ⅡA to heal cardiac damage were validated by the outcomes of cell viability, morphology, and proliferation. The efficacy of Tanshinone ⅡA in treating MIRI was further confirmed by the scratch assay and biomarker measurement. The differentially expressed genes were examined using transcriptome sequencing. The Ataxia-Telangiectasia Mutated (ATM)/Growth Arrest and DNA Damage (GADD45)/Origin Recognition Complex (ORC) signaling pathway was identified as being crucial to this process by KEGG pathway analysis and GO enrichment. Molecular docking and RT-qPCR were used to confirm our results. The crucial function of the ATM/GADD45/ORC pathway was further confirmed by the addition of an ATM inhibitor, which inhibited the expression of ATM.

**Conclusion:**

Tanshinone ⅡA can relieve the myocardial ischemia-reperfusion injury in cardiac cells by activating the ATM/GADD45/ORC pathway.

## 1 Introduction

Acute myocardial infarction (AMI) is an ischaemic heart disease caused by myocardial ischaemia and necrosis due to acute coronary artery congestion. It has become one of the greatest causes of mortality worldwide. Reperfusion, one of the most effective treatments for AMI, can help patients restore their coronary artery blood flow. However, increasing evidence shows that reperfusion treatment can cause myocardial ischemia-reperfusion injury (MIRI). MIRI has a complex pathomechanism involving metabolic disorders (Salameh et al., 2020), mitochondrial dysfunction (Tian et al., 2023), inflammation (Salameh et al., 2020; Tian et al., 2023), autophagy disorders and the release of high levels of reactive oxygen species (ROS) caused by oxidative stress (Yu et al., 2006). These injuries lead to myocardial damage and then aggravate heart failure or even lead to sudden cardiac death.

Among them, the excess ROS can attack DNA directly, causing base oxidation, glycation damage and DNA strand breaks. Massive release of inflammatory factors not only can increase oxidative stress but promote the expression of genes related to DNA damage. Furthermore, metabolic disorders can lead to a plunge in intracellular ATP levels, affecting the activity of DNA repair enzymes, exacerbating the consequences of DNA damage.

The ATM signaling pathway is a crucial signal transduction pathway that cells utilize to respond to DNA damage, particularly DNA double-strand breaks. Studies have shown that ATM activation can lead to Murine Double Minute 2 (MDM2) E3 phosphorylation and activity inhibition, which causes rapid accumulation of p53 (Chen, 2012). P53 activation can inhibit cell cycle progression (Chen, 2016). The cells arrest in the G2/M phase, and checkpoints are activated (St Clair et al., 2004). This arrest helps repair DNA damage, especially fatal double-strand breaks (Gatz and Wiesmüller, 2006).

Danshen is a traditional Chinese herb derived from the root and rhizome of Salvia miltiorrhiza, a Labiatae plant (Yuan et al., 2024). It promotes blood circulation to prevent blood stasis (Yuan et al., 2024; Cao et al., 2017). Danshen is frequently utilized in the clinical management of cardiovascular and cerebrovascular conditions (Qin et al., 2023). It can effectively relieve the clinical symptoms of ischemic cardiovascular and cerebrovascular diseases and reduce complications and sequelae.

Tanshinone ⅡA, the main liposoluble constituent of Danshen (Yang et al., 2020), has garnered widespread attention for its efficacy in treating AMI. Studies have shown that Tanshinone ⅡA possesses significant anti-inflammatory, antioxidant, and anti-fibrotic effects in cardiovascular protection. Ren et al. (2010) found that Tanshinone IIA effectively suppresses the release of inflammatory factors following myocardial infarction by reducing the expression of monocyte chemoattractant protein-1 (MCP-1), thereby decreasing the infiltration of inflammatory cells. This anti-inflammatory property is also crucial in the treatment of MIRI, as post-reperfusion inflammation often leads to secondary myocardial damage.

In addition to its anti-inflammatory effects, Tanshinone IIA demonstrates robust antioxidant potential. In AMI models, Tanshinone IIA enhances the activity of antioxidant enzymes such as superoxide dismutase (SOD) and glutathione peroxidase (GSH-Px) and reduces the production of oxidative stress marker malondialdehyde (MDA), thereby alleviating oxidative stress-induced myocardial damage (Chen et al., 2021). During MIRI, the sudden increase in ROS directly damages myocardial cells activates inflammatory signaling pathways, such as the NF-κB pathway, promoting the release of inflammatory factors (Hu et al., 2015). Therefore, Tanshinone IIA may effectively interrupt the vicious cycle of MIRI injury by inhibiting both oxidative stress and inflammatory signaling pathways.

Myocardial fibrosis is a core manifestation of myocardial remodelling after AMI and MIRI and is a primary pathological mechanism leading to heart failure. During fibrosis, myocardial tissue gradually hardens due to the deposition of type I and III collagen, which impairs normal cardiac function. Gao et al. (2019) indicated that TIIA also has significant anti-fibrotic effects, inhibiting myocardial fibrosis by reducing the expression of TGF-β and α-smooth muscle actin (α-SMA). This effect has potential application value in MIRI models, helping to prevent the progression of myocardial fibrosis and protecting cardiac function after myocardial infarction.

Previous studies have sufficiently demonstrated the therapeutic effects of Tanshinone IIA on AMI and MIRI. Therefore, we aim to investigate the regulatory role of Tanshinone on the ATM signaling pathway from the perspective of DNA damage, as well as the therapeutic mechanism in H9C2 cell ischemia-reperfusion injury. It provides experimental evidence supporting the usage of Tanshinone IIA in the prevention and treatment of ischemic cardiovascular diseases.

## 2 Materials and methods

### 2.1 Materials

The rat-myocardium-derived cardiac myoblast cell line H9C2 was purchased from the Union Medical University Cell Experiment Center (lot No. SCSP-5211).

Tanshinone ⅡA was purchased from the National Institutes for Food and Drug Control (lot No. WVH2-4LD1).

### 2.2 Cell culture and treatment

The rat cardiomyocytes, H9C2 cell line was cultivated in high-glucose DMEM (Gibco™, lot No. 8122512) supplemented with 10% (v/v) FBS (Invitrogen™, lot No. 10099-141). The culture was then incubated at 37°C with 5% CO_2_. Several groups were created out of these cells: normal group, model group and Tanshinone ⅡA treatment groups.

For the normal group, the cells were cultured and incubated as normal. For the model group, the cells were first treated as described for the normal group. PBS was used to wash the cells once the density reached 80%. The glucose-free DMEM (Gibco™, lot No.2323012) without serum was introduced. For 4 h, the cells were incubated at 37°C with 85% N_2_, 10% H_2_, and 5% CO_2_. Then, the medium was replaced with high-glucose DMEM, and the cells were incubated with 5% CO_2_ at 37°C for 2 h.

For the treatment groups, the cells were treated as the model group. Different doses of Tanshinone ⅡA were added to the serum and glucose-free DMEM to final concentrations of 1, 5, 10, 20, 40, 80, 160, 320, and 640 μmol/L.

For the inhibitor group, the ATM kinase inhibitor KU-55933 (Selleck, lot No. S1092) was added to the serum and glucose-free media together with Tanshinone ⅡA to a final concentration of 10 μmol/L.

### 2.3 CCK-8 assay

Approximately 10⁴ cells were added to each well of a 96-well plate. After they reached 80%–90% confluence, the cells were cultured as described in Section 2.2. Each well received 10 μL of CCK-8 solution (DOJINDO, lot No. TR689), and the plate was incubated for an hour with 5% CO_2_ at 37°C. The absorbance was measured following incubation at a wavelength of 450 nm.

### 2.4 Cellular morphology

The morphology of the cultivated cells was observed using an inverted microscope (Olympus Corporation, TH4-200).

### 2.5 Fluorescence staining

In a 6-well plate, about 2 × 10⁵ cells were put into each well. Following the outline described in Section 2.2, the cells were grown until they reached 80%–90% confluence. Culture media was used to dilute the EdU. The medium was supplemented with prewarmed EdU solution until the final concentration of EdU was 10 μmol/L. The cells underwent incubation in a CO_2_ environment at a temperature of 37°C for 2 h. Subsequently, the medium was substituted with a stationary medium, and the cells were cultured at room temperature for 15 min. Subsequently, the cells underwent a gentle wash with PBS, followed by the addition of 0.3% Triton X-100 to the cell suspension. The cells were cultivated for 10 min at room temperature, followed by a PBS wash and a further 20 min at room temperature with the blocking solution. Following washing, the cells were grown for 30 min at room temperature in the dark with 500 μL of Click solution added. Following a PBS wash, the cells were incubated for an additional 30 min at room temperature using 200 μL of streptavidin-HRP working solution. 200 μL of DAB colouring solution was applied after washing. After 10 min of RT culture, the cells were cleaned with PBS. DAPI was employed as the counterstain. A fluorescence microscope was used to observe and record the data. ImageJ was used to analyse the results.

### 2.6 Scratch assay

The 6-well plate was filled with about 10⁵ H9C2 cells per well, and the cells were cultivated until they achieved 80%–90% confluence. The cells were subsequently incubated as described in Section 2.2. A vertical line scratch was made in the centre of each well using a sterile 200 μL pipette tip. The wells were washed gently with PBS to remove detached cells and debris. Images of the scratches were captured with an inverted microscope. The cells were subsequently incubated with serum-free medium in 5% CO_2_ at 37°C. Images were captured every 12 h. ImageJ was used to analyse these images.

### 2.7 Determination of cellular supernatant biomarkers

The cellular supernatant was isolated to measure the levels of Aspartate transaminase (AST), Creatine kinase (CK) and Lactate dehydrogenase (LDH) via ELISA (Jianglai, lot No. JL21297-96T, JL46377-96T, JL46377-96T). The cells were subsequently centrifuged at 2,500 rpm and 4°C for 10 min, after which the supernatant was collected according to the kit instructions.

### 2.8 Transcriptomic sequencing

The cells underwent a washing procedure using pre-cold PBS, followed by total RNA extraction utilizing TRIzol. Oligo (dT) Dynabeads were employed for the isolation of mRNA. A reverse transcription kit was employed to synthesize the first-strand cDNA. Polymerase Chain Reaction (PCR) was subsequently used for cDNA amplification and cDNA library establishment. The Illumina sequencing platform was used to perform second-generation double-terminal sequencing.

### 2.9 RT‒qPCR

TRIzol was employed to extract the total RNA from the cells. The extracted RNA was utilized for the synthesis of cDNA through the process of reverse transcription. Then, the PCR solution system was prepared, split into microcentrifuge tube and amplified in a real-time quantitative PCR instrument.

The PCR solution system employed consisted of the following components: Hieff qPCR SYBR Green Master Mix (10 μL), forward primer (0.4 μL), reverse primer (0.4 μL), cDNA (2 μL), and ddH_2_O (7.2 μL). The sequences of the forward primer and reverse primer are presented in Table 1. GAPDH served as the reference gene. The 2^−ΔΔCT^ method was employed to determine the relative expression levels of mRNAs.

**TABLE 1:** PCR primer sequences.

Gene	Primer sequence (5′∼3′)	Product length/bp
ATM	F: CGA​GGC​GTA​CAA​TGG​TGA​AGG​AC	118
	R: TGG​TTG​GCT​GGA​ATG​CTG​ATG​C	
GADD45	F: CCG​CAG​AGC​AGA​AGA​TCG​AAA​GG	104
	R: CTC​GTA​CAC​GCC​GAC​AGT​TAT​GG	
ORC	F: GGA​GCA​CAG​CAG​AAC​AAG​AGG​TC	119
	R: ACC​ACC​ACA​TTC​CAG​AGA​CTT​CAT​TG	
GADPH	F: TCT​GAG​CCT​CCT​CCA​ACC​CAA​C	108
	R: CGT​TCA​CAC​CGA​CCT​TCA​CCA​TC	

The reaction conditions were established as follows: an initial denaturation phase at 95°C for a duration of 10 min, followed by denaturation at 95°C for 10 s, and an annealing phase at 60°C for 30 s. Denaturation and annealing were regarded as one cycle. Forty cycles were performed in this reaction.

### 2.10 Molecular docking

The three-dimensional structural files of the target proteins were acquired from the Protein Data Bank (https://www.rcsb.org/) and UniProt (https://www.uniprot.org/) databases. The molecular structures of the ligands were obtained from the PubChem database (https://pubchem.ncbi.nlm.nih.gov/). Coordinate files for the receptor and ligand were generated utilizing AutoDock Tools software. The receptor underwent the removal of water molecules, followed by the separation of the ligand from the receptor. Following the incorporation of nonpolar hydrogens, molecular docking was performed (Cao et al., 2023).

### 2.11 Western blot

The cells were harvested and subjected to lysis with a suitable lysis buffer that included protease inhibitors, followed by the assessment of protein concentration utilizing a BCA kit. Equal volumes of protein were combined with loading buffer, subjected to boiling at 95°C for 5 min, subsequently loaded into 8% SDS-PAGE gels, and then electrophoresed to achieve separation of proteins according to their molecular weight. The proteins were subsequently transferred onto a PVDF membrane utilizing wet transfer techniques. The membrane underwent a blocking procedure using a blocking buffer composed of 5% non-fat milk in TBST for a duration of 90 min at room temperature, aimed at minimizing non-specific binding. The samples were incubated overnight at 4°C with primary antibodies targeting ATM, GADD45, ORC, and β-actin (Cell Signalling, 2,873, 4,632, 4,731, 4,967), which served as the internal control, all diluted in blocking buffer at the concentration of 0.5 μL/mL. Following the washing procedure, the membrane underwent incubation with HRP-conjugated secondary antibodies, which were diluted in blocking buffer for 1 h at room temperature. The membrane underwent a subsequent washing, followed by the application of enhanced chemiluminescence (ECL) substrate, and the detection of protein bands was accomplished utilizing a chemiluminescence imaging system. The analysis of band intensities was conducted utilizing ImageJ for quantification.

### 2.12 Statistical analysis

For data analysis, GraphPad Prism 10.1.2 was utilised. The results were presented as mean ± standard deviation (mean ± sd). One-way ANOVA was employed to examine the differences between the two groups. When p < 0.05, the differences were considered statistically significant.

## 3 Results

### 3.1 Effect of Tanshinone ⅡA on MIRI in the H9C2

**FIGURE 1:**
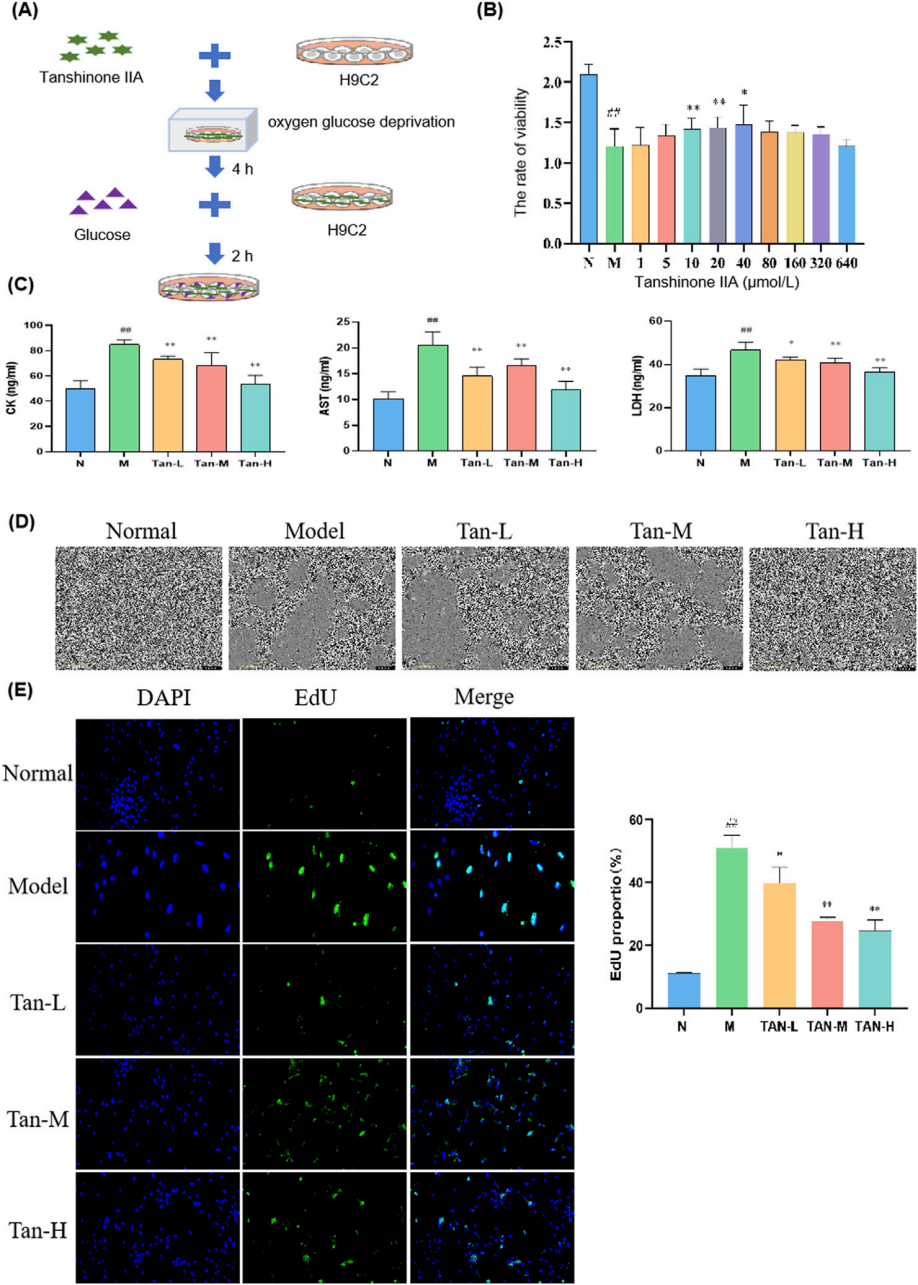
Effect of Tanshinone ⅡA on myocardial ischemia-reperfusion injury in the H9C2. Notes: **(A)** Cell culture and treatment; **(B)** viability rate of H9C2; **(C)** changes in the level of CK, AST and LDH in H9C2; **(D)** morphology of H9C2 cells; **(E)** fluorescence staining of H9C2 cells. ^##^ *p* < 0.01 vs. the normal group, ^*^ *p* < 0.05 vs. the model group, ^**^ *p* < 0.01 vs. the model group (n = 6, Mean ± sd).

Cells in Tanshinone ⅡA treatment groups were treated as shown in Figure 1A. The model group exhibited a significant reduction in cell viability when compared to the normal group (p < 0.01). In contrast, the treatment groups receiving Tanshinone IIA exhibited a notable increase in cell viability at concentrations of 10 and 20 μmol/L (p < 0.01) and 40 μmol/L (p < 0.05) in comparison to the model group (Figure 1B).

Based on these cell viability results, concentrations of 10, 20, and 40 μmol/L were selected for further experiments. Figure 1C illustrates that the levels of CK, AST, and LDH were markedly elevated in the model group compared to the normal group (p < 0.01). In comparison to the model group, the levels of CK and AST were markedly reduced in the Tan-L (10 μmol/L), Tan-M (20 μmol/L), and Tan-H (40 μmol/L) groups (p < 0.01). Comparably, the levels of LDH exhibited a significant decrease in the Tan-L group (p < 0.05), Tan-M group (p < 0.01), and Tan-H group (p < 0.01) when contrasted with the model group.

Figure 1D shows that cells in the normal group were neatly arranged with regular shapes and small intercellular spaces. In contrast, cells in the model group varied in size, were loosely arranged with large intercellular spaces, and exhibited numerous pieces of cell debris. The Tan-L and Tan-M groups displayed more closely arranged cells with relatively regular shapes, though some irregular areas remained. Cells in the Tan-H group were tightly arranged with regular shapes and showed only mild signs of damage.

According to the DAPI and EdU staining results presented in Figure 1E, Tanshinone IIA treatment effectively alleviated the decrease in cell viability and abnormal proliferation caused by the model.

As indicated in Table 2, the relative wound density in the model group was significantly lower than that of the normal group (p < 0.01). Compared to the model group, the relative wound density significantly decreased at 60 h in both the medium-dose and high-dose Tanshinone IIA treatment groups (p < 0.01).

**TABLE 2:** Relative wound density (mean ± sd).

Group	Dose (μmol/L)	n	12 h (%)	24 h (%)	36 h (%)	48 h (%)	60 h (%)
Normal	—	5	42.06 ± 1.82	59.26 ± 2.29	71.83 ± 1.83	77.94 ± 1.99	85.21 ± 1.44
Model	—	5	5.97 ± 1.05^##^	6.89 ± 1.39^##^	9.75 ± 1.52^##^	13.31 ± 1.59^##^	16.62 ± 1.66^##^
Tan-L	10	5	8.20 ± 0.68	9.42 ± 0.96	12.65 ± 1.57	15.83 ± 1.58	19.16 ± 1.67
Tan-M	20	5	7.46 ± 1.02	8.21 ± 0.92	10.14 ± 1.10	14.93 ± 1.71	21.70 ± 3.29^**^
Tan-H	40	5	11.18 ± 2.08^**^	14.11 ± 3.16^**^	17.96 ± 3.91^**^	21.17 ± 4.43^**^	28.27 ± 4.27^**^

Note: ^##^ *p* < 0.01 vs. the normal group, ^**^ *p* < 0.01 vs. the model group.

### 3.2 Gene differential expression analysis via transcriptomic sequencing

**FIGURE 2:**
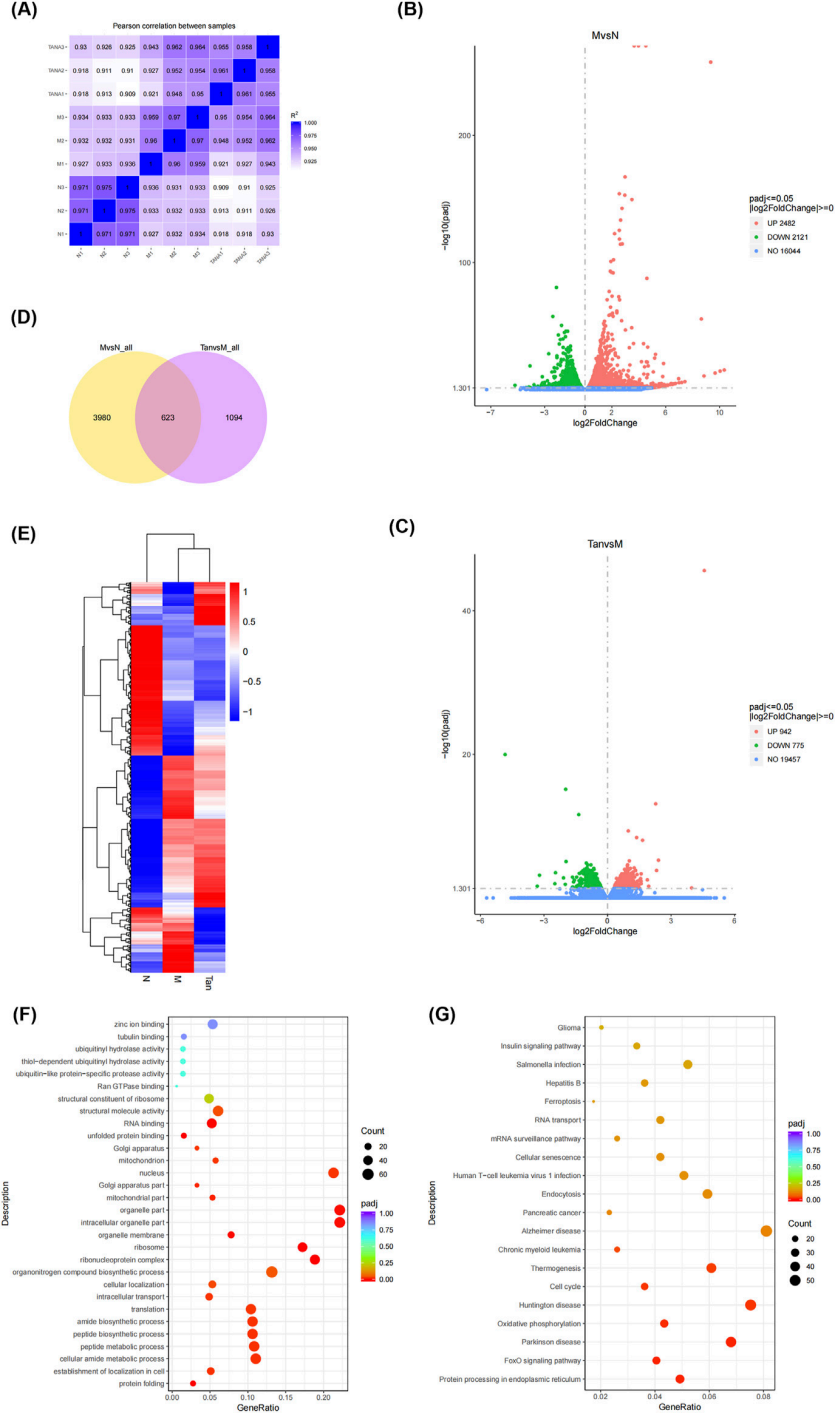
Gene differential expression analysis. Notes: **(A)** Gene correlation analysis between groups; **(B)** volcano plot of differentially expressed genes in the model group and normal group; **(C)** volcano plot of differentially expressed genes in the Tanshinone ⅡA treatment group and model group; **(D)** venn diagram of differentially expressed genes in the groups; **(E)** clustering heatmap of differentially expressed genes; **(F)** GO enrichment analysis; **(G)** KEGG pathway analysis.

A correlation heatmap (Figure 2A) was used to visualize gene correlations between the groups. It revealed that all Pearson coefficients were greater than 0.9, indicative of strong positive correlations.

The gene with a p-value threshold of less than 0.05 and a log_2_(FC) threshold less than or more than 0 was identified as a differentially expressed gene. Differentially expressed genes are illustrated in volcano plots (Figures 2B, C). In comparison to the control group, the experimental group demonstrated 4,603 differentially expressed genes, comprising 2,482 genes that were upregulated and 2,121 genes that were downregulated (Figure 2B). In the analysis of the Tanshinone IIA treatment group versus the model group, a total of 1,717 differentially expressed genes were identified, comprising 942 that were upregulated and 775 that were downregulated (Figure 2C).

A Venn diagram (Figure 2D) visualizes the distribution and overlap of the differentially expressed genes. Of the 4,603 genes differentially expressed between the normal and model groups and the 1,717 genes between the model and Tanshinone IIA treatment groups, 623 genes were commonly differentially expressed.

As shown in Figure 2F, the differentially expressed genes were enriched in Gene Ontology categories encompassing biological processes (BP), cellular components (CC), and molecular functions (MF). The main biological processes involved protein folding, peptide synthesis, and translation. The cellular components primarily included the ribosome, mitochondrial parts, and nucleus. Key molecular functions were unfolded protein binding, RNA binding, and structural molecule activity. The KEGG pathway analysis (Figure 2G) revealed that these genes were predominantly enriched in pathways associated with the cell cycle, Huntington’s disease, oxidative phosphorylation, Parkinson’s disease, the FoxO signaling pathway, and protein processing in the endoplasmic reticulum.

### 3.3 Tanshinone ⅡA relieves myocardial ischemia-reperfusion injury through ATM/GADD45/ORC pathway activation

**FIGURE 3:**
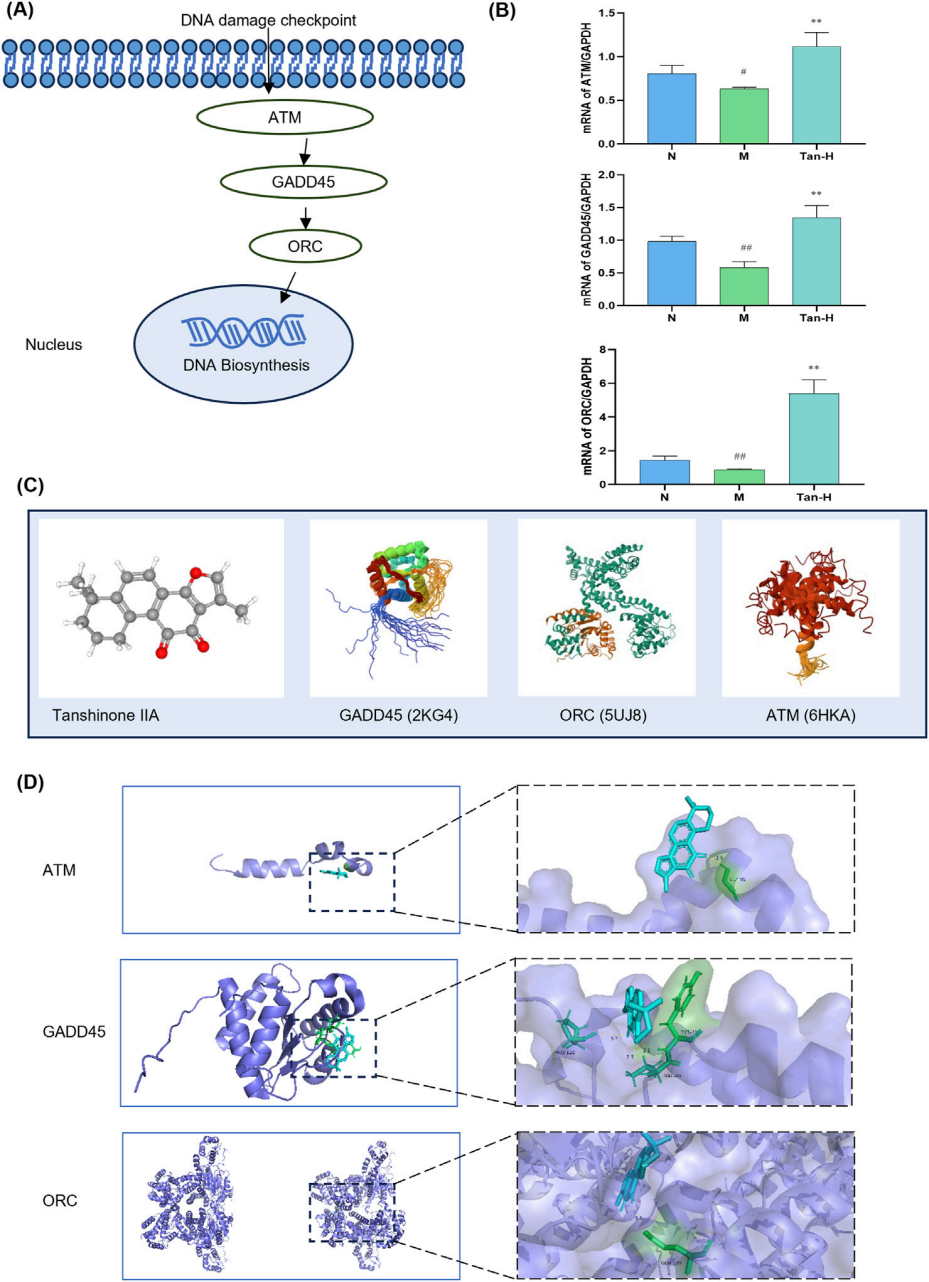
Pathway validation. Notes: **(A)** ATM/GADD45/ORC pathway; **(B)** mRNA expression of ATM, GADD45 and ORC in H9C2; **(C)** molecular structure of Tanshinone ⅡA and pathway-related proteins; **(D)** molecular docking image of Tanshinone ⅡA and pathway-related protein. ^#^ *p* < 0.05 vs. the normal group, ^##^ *p* < 0.01 vs. the normal group, ^**^ *p* < 0.01 vs. the model group (n = 6, mean ± sd).

Based on the KEGG pathway analysis and literature review, the cell cycle has been identified as a key pathway through which Tanshinone IIA alleviates myocardial ischemia-reperfusion injury. As a result, the pathway genes in the cell cycle were imported into Cytoscpe. The core genes were screened using CytoHubba, and the top three genes, ATM,GADD45 and ORC, were selected for RT-qPCR analysis according to the Degree value. To verify the effectiveness of ATM/GADD45/ORC pathway (Figure 3A), follow-up experiments were conducted.

Figure 3B illustrates that the model group exhibited a notable reduction in ATM expression relative to the normal group (p < 0.05), alongside significant decreases in GADD45 and ORC expressions (p < 0.01). Moreover, the Tan-H group demonstrated significantly reduced expression levels of ATM, GADD45, and ORC compared to the model group (p < 0.01).

Figure 3C presents the molecular structure of Tanshinone IIA alongside the 3D structures of GADD45, ORC, and ATM. The docking results in Figure 3D indicate that Tanshinone IIA can interact with GADD45, ORC, and ATM. Specifically, it binds to ATM and ORC via hydrogen bonds with GLY-95 and GLN-399, respectively, and forms three hydrogen bonds with GADD45 involving VAL-40, TYR-41, and PRO-120.

**FIGURE 4:**
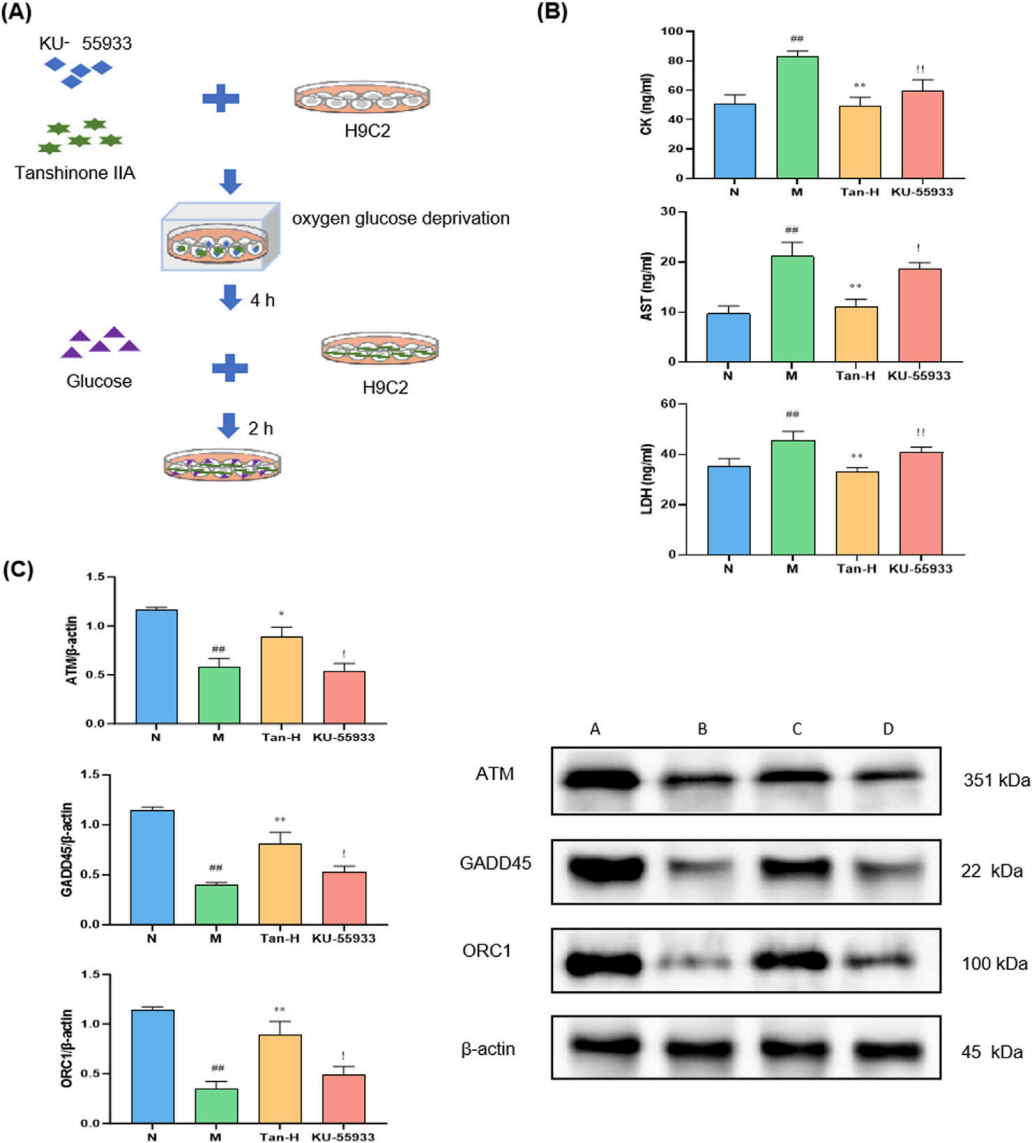
Pathway validation through ATM inhibitor. Notes: **(A)** Cell culture and treatment; **(B)** changes in the level of CK, AST and LDH in H9C2; **(C)** expression of ATM, GADD45 and ORC in H9C2. ^#^ *p* < 0.05 vs. the normal group, ^##^ *p* < 0.01 vs. the normal group; *p < 0.05 vs. the model group, ^**^ *p* < 0.01 vs. the model group; ^!^ *p* < 0.05 vs. the Tanshinone ⅡA treatment group, ^!!^ *p* < 0.01 vs. the Tanshinone ⅡA treatment group (n = 6, mean ± sd).

To further validate the involvement of the ATM/GADD45/ORC pathway, the ATM inhibitor KU-55933 was introduced into the model system (Figure 4A).

Figure 4B illustrates that the model group demonstrated markedly elevated levels of CK, AST, and LDH in comparison to the normal group (p < 0.01). Conversely, the Tan-H group exhibited markedly lower levels of these enzymes in comparison to the model group (p < 0.01). In comparison to the Tan-H group, the inhibitor group exhibited a statistically significant elevation in CK levels (p < 0.05) and notably higher concentrations of AST and LDH (p < 0.01).

Figure 4C demonstrates that the model group exhibited markedly reduced expression levels of ATM, GADD45, and ORC1 in comparison to the normal group (p < 0.05). The Tan-H group demonstrated a notable enhancement in the expression of these genes in comparison to the model group (p < 0.05). Nonetheless, the inhibitor group exhibited markedly lower expression levels of ATM, GADD45, and ORC1 in comparison to the Tan-H group (p < 0.05).

## 4 Discussion

Acute myocardial infarction is a global ischemic cardiovascular disease with high mortality and morbidity (Li et al., 2023). As of 2020, approximately 1.72% of the global population was plagued with AMI (Khan et al., 2020). Reperfusion therapy is an effective treatment for ischemic diseases. However, ischemia-reperfusion injury after reperfusion is also fatal (Verma et al., 2002). As a result, prevention and treatment of MIRI have become a key procedure during AMI treatment. It always causes myocardial necrosis, endothelial dysfunction, inflammation and oxidative stress (Yao et al., 2023).

Pharmacological studies have shown that Danshen promotes coronary artery expansion, improves myocardial ischemia and repairs myocardial damage. It can also increase the hypoxia tolerance of myocardial cells, thereby protecting the anoxic myocardium (Feng et al., 2022). The effective constituent Tanshinone ⅡA has the effects of heart protection, coronary dilation, anti-atherosclerosis, anti-platelet, anticoagulant, anti-thrombosis and inhibition of myocardial hypertrophy (Cao et al., 2017; Feng et al., 2022).

According to the previous research, Tanshinone ⅡA has a great therapeutic effect to MIRI. Hu et al. used rat cardiomyocytes, H9C2, to establish MIRI cell model (Hu et al., 2023) and found that Tanshinone ⅡA effectively attenuated H9c2 cardiomyocyte damage through inhibiting ferroptosis in the MIRI injury model. Li et al. used SD rats to establish MIRI rat model (Li et al., 2016) and found that Tanshinone IIA could activate the PI3K/Akt/mTOR signaling pathway to relieve MIRI in rats. Pan et al. used C57BL/6 mice to establish MIRI mouse model (Zhai et al., 2024) and found that Tanshinone IIA contributed to the improvement of the area of myocardial infarction, reducing myocardial enzyme levels, and promoting myocardial contractility recovery. Liu et al. used Japanese white rabbits to establish MIRI rabbit model (Liu et al., 2017) and found that Tanshinone IIA can significantly enhance the secretion of VEGF and the activity of oxygen free radical scavenging enzymes, accelerate the neovascularization of vascular endothelium in ischemic myocardium, promote angiogenesis, inhibit lipid peroxidation reactions during MIRI and alleviate the inflammatory response of myocardial vascular endothelium. Thus, it improves the function of damaged vascular endothelium after I/R injury and has a definite protective effect on acute MIRI in rabbits. Researchers have used various cells and experimental animals to establish different models of MIRI, and all have shown that Tanshinone IIA has a preventive and therapeutic effect on MIRI. It is considered that the effect of Tanshinone IIA to this disease model has been proved and there is no need to add a positive drug to certify its therapeutic effect. Besides, the mechanisms of commonly used positive drugs in ischemic cardiovascular disease are not accordant with the mechanism studied in the research. As a result, the positive control was not mentioned in the whole study.

In this study, the myoblast cell line H9C2 was deprived of oxygen and glucose to establish an MIRI model *in vitro*. Tanshinone ⅡA was added at the same time to simulate MIRI. In terms of cell viability, morphology and migration, significant differences were detected between the normal group and the model group, indicating the success of the cell model. A statistically significant difference existed between the model group and the Tanshinone ⅡA treatment groups. These findings suggest that Tanshinone ⅡA can reduce myocardial apoptosis caused by MIRI. In addition, the EdU staining results revealed that Tanshinone ⅡA treatment relieved the decrease in myocardial cell viability and abnormal proliferation caused by MIRI.

After modelling, cardiac biomarkers were measured to verify the effect of Tanshinone ⅡA on myocardial protection from MIRI. CK, AST and LDH are cardiac markers commonly used in the clinic. The expression levels can be used to quantify the extent of MIRI (Toldo et al., 2018). In this study, the levels of CK, AST and LDH decreased, and the cell viability increased significantly after treatment. These findings suggest that Tanshinone ⅡA may reduce inflammation, improve myocardial cell apoptosis and thereby protect against MIRI.

Then, transcriptomic sequencing was performed to analyse the differences in gene expression between the groups. According to the transcriptomic sequencing results, many genes exhibited differences among the groups. Many genes were enriched in the cell cycle pathway. ATM, GADD45 and ORC are key genes in the cell cycle pathway, and their transcriptomic sequencing results also differ greatly.

**FIGURE 5:**
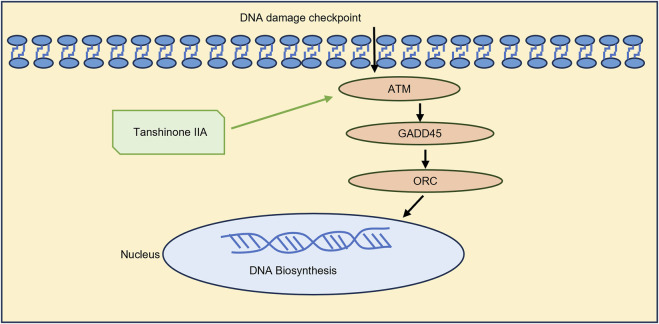
Mechanism by which Tanshinone ⅡA improves myocardial damage caused by MIRI.

The RT‒qPCR results revealed that Tanshinone ⅡA can increase the mRNA expression of ATM, GADD45 and ORC in cardiomyocytes damaged by MIRI. On the basis of the gene differential analysis and RT‒qPCR results, we can speculate that Tanshinone ⅡA can repair DNA damage and promote DNA biosynthesis by stimulating key factors involved in the ATM/GADD45/ORC pathway (Figure 5). The molecular docking results revealed the combination of Tanshinone ⅡA with these three proteins, which may provide additional evidence supporting the above conjecture.

ATM, GADD45 and ORC are key proteins involved in ATM signaling pathway. ATM is a serine‒threonine protein kinase. It is the key factor in the DNA damage response. It can phosphorylate p53 and Chk2 (Li and Stern, 2005; Meulmeester et al., 2005). Therefore, the interaction between MDM2 and p53 is inhibited, which can increase p53 levels, inhibit the cell cycle, and trigger checkpoint and DNA repair activation (Kastan and Lim, 2000). P53 can bind with GADD45 in the promoter region and transcriptionally activate it (Kim et al., 2013). GADD45 is also a crucial protein in DNA repair. It can stimulate p38-JNK mitogen-activated protein kinase (MAPK), mediating the immune response (Rodríguez-Jiménez et al., 2021). GADD45 expression is related to ROS production and the activation of NADPH oxidase, which potentially promotes the counter-regulation of oxidative damage (Wan et al., 2000; De La Fuente et al., 2009). On the other hand, active Chk2 can amplify DNA damage signals (Li and Stern, 2005). Its key substrate, CDC25A, can activate CDK2 (Ditano et al., 2021), thereby activating ORC. The ORC is the origin of DNA replication and can regulate the cell cycle (Ohta et al., 2003). The increased expression of these three genes can improve DNA repair and new DNA biosynthesis. To verify the above findings, an ATM inhibitor was used for cell treatment. KU-55933 was used in cell culture together with Tanshinone ⅡA to inhibit the expression of ATM. The levels of CK, AST, and LDH in the cell supernatant of the inhibitor group were markedly elevated compared to those observed in the Tan-H group. Furthermore, the Western blot analysis demonstrated that Tanshinone ⅡA markedly enhanced the protein expression levels of ATM, GADD45, and ORC1 through ATM activating.

The experimental findings indicate that Tanshinone ⅡA mitigates myocardial ischemia-reperfusion injury in cardiac cells. The mechanism could be linked to the activation of the ATM/GADD45/ORC pathway.

## Data Availability

The data presented in the study are deposited in Zenodo, accession DOI: 10.5281/zenodo.14284052. It can be accessed through the URL: https://zenodo.org/records/14284052.
